# Molecular Characterization of Escherichia coli Causing Urinary Tract Infections Through Next-Generation Sequencing: A Comprehensive Analysis of Serotypes, Sequence Types, and Antimicrobial and Virulence Genes

**DOI:** 10.7759/cureus.55556

**Published:** 2024-03-05

**Authors:** Venkataramana Kandi, Praveen R Shahapur, Tarun Kumar Suvvari, Vallab Ganesh Bharadwaj, Chitra Rajalakshmi P, Roopa Shahapur, Eswar Podaralla, Vikram Godishala

**Affiliations:** 1 Clinical Microbiology, Prathima Institute of Medical Sciences, Karimnagar, IND; 2 Microbiology, Bijapur Lingayat District Educational (BLDE) (Deemed to be) University, Shri B. M. Patil Medical College, Vijayapura, IND; 3 General Medicine, Rangaraya Medical College, Kakinada, IND; 4 Research, Squad Medicine and Research (SMR), Visakhapatnam, IND; 5 Microbiology, Trichy Sri Ramaswamy Memorial (SRM) Medical College Hospital and Research Centre, Tiruchirappalli, IND; 6 Dentistry, Bijapur Lingayat District Educational (BLDE) (Deemed to be) University, Shri B. M. Patil Medical College, Vijayapura, IND; 7 Internal Medicine, Temple University, Philadelphia, USA; 8 Biotechnology/Food and Nutrition, Vaagdevi Degree and PG College, Hanamkonda, IND

**Keywords:** virulence genes, extended-spectrum beta-lactamases (esbls), drug-resistant bacterial strains, resistance genes, multilocus sequence typing (mlst), escherichia coli (e. coli), whole genome sequencing (wgs), antimicrobial resistance (amr)

## Abstract

Introduction

An enormous increase in antimicrobial resistance (AMR) among bacteria isolated from human clinical specimens contributed to treatment failures. Increased surveillance through next-generation sequencing (NGS) or whole genome sequencing (WGS) could facilitate the study of the epidemiology of drug-resistant bacterial strains, resistance genes, and other virulence determinants they are potentially carrying.

Methods

This study included 30 *Escherichia coli* (*E*. *coli*) isolates obtained from patients suffering from urinary tract infections (UTIs) attending Prathima Institute of Medical Sciences, Karimnagar, India. All bacterial isolates were identified, and antimicrobial susceptibility patterns were determined through conventional microbiological techniques and confirmed by automated systems. All the isolates were investigated using NGS to identify genes coding for resistance, such as extended-spectrum beta-lactamases (ESBLs), metallo-beta-lactamases, and virulence genes. Multilocus sequence typing (MLST) was used to understand the prevalent strain types, and serotyping was carried out to evaluate the type of O (cell wall antigen) and H (flagellar antigen) serotypes carried by the isolates.

Results

The conventional antimicrobial susceptibility testing revealed that 15 (50%) isolates were resistant to imipenem (IPM), 10 (33.33%) were resistant to amikacin (AK), 13 (43.33%) were resistant to piperacillin-tazobactam (PTZ), 17 (56.66%) were resistant to cephalosporins, and 14 (46.66%) were resistant to nitrofurantoin (NIT). Among the isolates, 26 (86.66%) had revealed the presence of multiple antibiotic-resistant genes with evidence of at least one gene coding for beta-lactamase resistance. There was a high prevalence of *bla*_CTX-M _(19/30, 63.33%) genes, followed by *bla*_TEM_ and *bla*_OXA-1_. The *bla*_NDM-5_ gene was found in three isolates (3/30, 10%). The virulence genes identified in the present study were *iutA*, *sat*, *iss*, and *papC*, among others. The *E*. *coli* serotype found predominantly belonged to O25:H4 (5, 16.66%), followed by O102:H6 (4, 13.33%). A total of 16 MLST variants were identified among the examined samples. Of the MLST-based sequence types (STs) identified, ST-131 (7, 23.33%) was the predominant one, followed by ST-167 (3, 10%) and ST-12 (3, 10%).

Conclusions

The study results demonstrated that the *E*. *coli *strains isolated from patients suffering from UTIs potentially carried antimicrobial resistance and virulence genes and belonged to different strain types based on MLST. Careful evaluation of bacterial strains using molecular analyses such as NGS could facilitate an improved understanding of bacterial antibiotic resistance and its virulence potential. This could enable physicians to choose appropriate antimicrobial agents and contribute to better patient management, thereby preventing the emergence and spread of drug-resistant bacteria.

## Introduction

Antimicrobial resistance (AMR) is the ability of bacteria to resist its clearance from the hosts despite treatment. Bacteria resistant to various antimicrobial agents are labeled multidrug-resistant (MDR) bacteria. There are several mechanisms by which bacteria develop AMR, including mutations, drug efflux mechanisms, and alteration of drug-binding proteins, among others. Additionally, bacteria employ different virulence mechanisms, such as adherence and biofilm formation, which enable them to counteract the activities of drugs. Moreover, AMR is coded by genes on the chromosomes and plasmids, which could be transmissible from one bacterium to another bacterium. This can be achieved through intraspecies (vertical gene transfer) and interspecies (horizontal gene transfer) genetic transfer mechanisms that occur naturally among bacteria. The excessive use of antimicrobial agents, especially among hospitalized patients, facilitates the development of resistant bacterial species. Besides, people resort to self-medication by using over-the-counter drugs, which results in resistance among the bacterial species prevailing in individuals and communities. Subsequently, these resistant bacterial species could be accountable for hospital-acquired infections (HAIs) and community-acquired infections (CAIs). There is an increased probability of the spread of such bacterial species from one person to another in the community, from patient to patient, and from healthcare workers (HCWs) to patients in hospital settings. Infections with drug-resistant bacteria result in treatment failures [1].

The emergence of AMR cannot be predicted and probably is difficult to prevent, as observed by a recent case study of a patient suffering from pancreatitis. This case study emphasizes the role of clinicians in discussing and evaluating antibiotic treatment, which otherwise would result in AMR and treatment failure. It was suggested that patients must be assessed for any preexisting AMR determinants in colonized bacteria. Screening for AMR among bacteria in the hospital environment and evidence of de novo resistance that may be responsible for horizontal gene transfer was also recommended [2].

*Escherichia coli *(*E*. *coli*) is a commensal bacteria present in the intestines of humans and animals. Apart from being a normal flora of the intestines, *E*. *coli *has been identified as the most predominant bacteria to cause urinary tract infections (UTIs). *Escherichia coli *strains with pathogenic potential are responsible for other infections such as toxin-mediated diarrhea, bloodstream infections, and wound infections. Further, neonates and other individuals with suppressed immune systems could be predisposed to various infections caused by *E*. *coli *[3]. Considering the complexity of this bacterium, as evidenced by its existence as a commensal, its presence in the environment, and its potential to cause mild to severe infections, it is essential to understand its epidemiological features, virulence determinants such as antimicrobial resistance capabilities, and invasive potential, among others. This study, therefore, is carried out to evaluate the presence of resistance and virulence genes in *E*. *coli *isolated from patients suffering from UTIs. Additionally, they were classified into serotypes and sequence types (STs) based on molecular characterization utilizing next-generation sequencing (NGS) or whole genome sequencing (WGS).

## Materials and methods

An observational, analytical, cross-sectional study was conducted among patients attending Prathima Institute of Medical Sciences, Karimnagar, Telangana, India. This study included 30 *E*. *coli *isolates obtained from patients suffering from urinary tract infections from April 2018 to April 2020. All bacterial isolates were identified, and antimicrobial susceptibility patterns were determined through conventional microbiological techniques and confirmed by automated systems [4-6]. Further, all the isolates were analyzed using NGS/WGS to identify the genes coding for drug resistance and virulence. Multilocus sequence typing (MLST) was used to understand the prevalent strain types, and serotyping was carried out to evaluate the type of O (cell wall antigen) and H (flagellar antigen) serotypes expressed by the isolates.

Processing of urine samples

**Figure 1:**
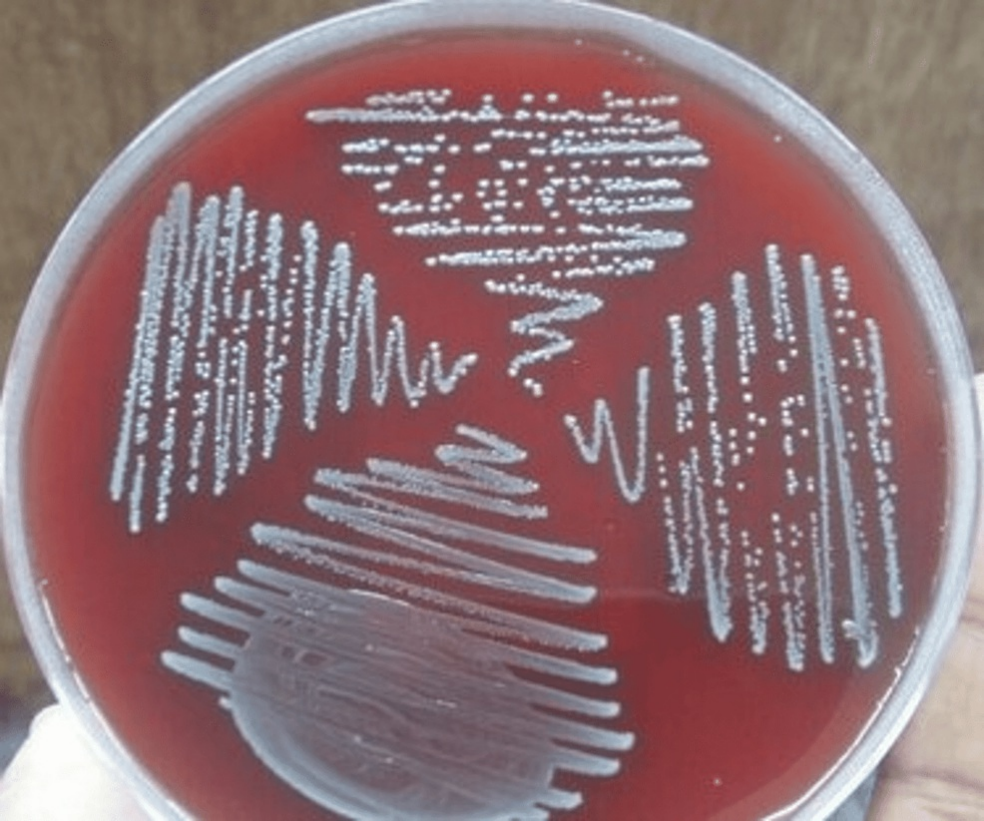
Growth obtained on blood agar from urine sample for semi-quantitative estimation of bacteria

A wide-mouthed sterile container was provided to the patient. The patient was advised to clean the genital areas with soap and water. Later, the patient was asked to collect mid-stream urine after passing out the initial urine. The patient was advised not to touch the inside of the container, and the urine should not overflow from the container. The sample container was appropriately labeled and was immediately transported to the laboratory for processing. When delays in transporting specimens to the laboratory are expected, the urine samples were refrigerated between 2°C and 8°C. The sample was processed by inoculating 0.001 milliliters (mL) of urine in blood agar. The method used here was the semiquantitative counting technique. In this method, the urine sample is plated on the culture media after dividing the plate into four quadrants, as shown in Figure 1.

After overnight incubation of the inoculated plates at 37°C, the growth was recorded by counting the number of colonies or presence of growth in different quadrants of blood agar. The patients whose urine contained ≥10^5^ colony-forming units (CFUs) per mL of urine were considered to be suffering from UTI. The CFUs are nothing but bacteria, i.e., each colony corresponds to a bacterium [7].

Interpretation

Growth in the first quadrant corresponds to 25,000 colonies, both in the first and second quadrants corresponds to 50,000 colonies, growth in the three quadrants corresponds to 75,000 colonies, and growth in all the quadrants corresponds to 100,000 or 10^5^ CFU/mL of urine (Figure 1).

Antibiotic susceptibility testing

Kirby-Bauer Disk Diffusion Method

Two to three pure and isolated colonies from overnight bacterial growth (inoculum) were picked up from the culture plate. The inoculum was mixed well into the peptone water/sterile saline. Later, it was incubated at 37°C for 1-2 hours. The test tube now shows growth in the form of turbidity. The turbidity is adjusted to match turbidity standards, as measured by the McFarland standards. The McFarland standards are used for measuring turbidity manually by comparing and adjusting the culture turbidity with a solution prepared by mixing 0.05 mL of 1% barium chloride and 9.95 mL of 1% sulfuric acid.

After adjusting to the desired McFarland standards, the test organisms were inoculated into Mueller-Hinton agar (MHA). A lawn culture/carpet culture was made with the help of sterile cotton swabs. Later, different antibiotic-impregnated filter paper disks were applied with the help of sterile forceps. The plates were then incubated overnight at 37°C for 12-18 hours. If an organism is not growing near the antibiotic discs, i.e., if organisms are sensitive/susceptible to the antibiotic, there is a zone of inhibition/clearance, measured in millimeters (mm). However, if the microorganisms are resistant to the antibiotic, growth will be noted even closer/toward the edge of the antibiotic-impregnated disk. The results were interpreted according to the Clinical and Laboratory Standards Institute (CLSI) guidelines [8]. Isolates with phenotypic resistance, including both resistant (R) or intermediate (I) resistance, are considered resistant (R). An isolate was designated as MDR when it showed resistance to more than one agent in three or more antimicrobial categories.

Antibiotics Tested

The following antibiotics were used: imipenem (IPM) (10 µg), amikacin (AK) (30 µg), gentamicin (GEN) (10 µg), ciprofloxacin (CIP) (5 µg), ofloxacin (OF), cotrimoxazole (COT) (1.25/23.75 µg), piperacillin-tazobactam (PTZ) (30/6 µg), ceftazidime (CAZ) (10 µg), ceftriaxone (CTR) (30 µg), cefotaxime (CTX) (30 µg), cephalothin (30 µg), and nitrofurantoin (NIT) (300 µg).

Control Strains

*Escherichia coli *American Type Culture Collection (ATCC) 25922, *Klebsiella pneumoniae *ATCC 1706, *Pseudomonas aeruginosa *ATCC 27853, and *Staphylococcus aureus *ATCC 25923 were used as controls.

Whole genome sequencing and genomic analyses

The deoxyribonucleic acid (DNA) was extracted from bacterial isolates using the QIAamp DNA Mini Kit (Qiagen, Hilden, Germany) as mandated by the manufacturer's instructions. Double-stranded DNA libraries with 450 base pairs (bp) insert size were prepared and sequenced on the Illumina platform with 150 bp paired-end chemistry. The genomes that passed sequence quality control were assembled using Spades v3.14 to generate contigs and annotated with Prokka v1.5 [9,10]. Species identification was carried out using a Bactinspector, and contamination was assessed using ConFindr [11]. All the quality metrics were combined using MultiQC and qualifyr to generate web-based reports. MLST, AMR, and virulence factors were identified using ARIBA tool v2.14.4 with BIGSdb-Pasteur MLST database, National Center for Biotechnological Information (NCBI) AMR acquired gene, PointFinder databases, and VFDB, respectively [12-14]. All bioinformatic analysis was performed using Nextflow pipelines developed as a part of the Global Health Research Unit (GHRU)-AMR.

## Results

The mean age of the patients was 46.96±20.18 years. Among the patients included, 19 (63.33%) were males, and 11 (36.66%) were females. The study identified several genes that could contribute to increased virulence and facilitate the colonization, adaptability, and spread of the bacteria inside humans. These genes enable the organisms to become increasingly invasive and produce pathogenic effects. Additionally, the study's results showed the presence of antibiotic-resistant genes that contribute to MDR. Details of the virulence genes and the antimicrobial resistance genes along with their functions are shown in Table 1.

**Table 1:** Antibiotic resistance and virulence gene along with their functions *bla*: beta-lactamase gene, MBL: metallo-beta-lactamase, RND: resistance-nodulation-cell division, *bla*_NDM_: New Delhi metallo-beta-lactamase, *Ble*: bleomycin gene, NADP: nicotinamide adenine dinucleotide phosphate, RTX: repeats in toxin, ATP: adenosine 5'-triphosphate, ABC: ATP-binding cassette, MFS: major facilitator superfamily, RNA: ribonucleic acid, SMR: small multidrug resistance, ESBLs: extended-spectrum beta-lactamases

Resistance/virulence genes	Function
*aac *(3IIe, 3IId, 6Ib, 6Ibcr5)	Aminoglycoside N-acetyltransferase
*aad *(A1, A2, A5)	Ant3 Ia family aminoglycoside nucleotidyltransferase
*aph *(3Ia, 3Ib, 6Id)	Aminoglycoside-o-phosphotransferase
arr1	Rifampin adenosine diphosphate-ribosyl transferase
*ble*EC	Cephalosporin resistance
*bla*_AFM-2_	Subclass 'B1' MBL
*bla*_CMY-2_, *bla*_CMY-4_	AmpC beta-lactamase
*bla*_CTX-M__-15_	Class 'A' ESBL
*bla*EC (5, 8, 15, 16, 18, 19)	Serine beta-lactamase with a substrate specificity for cephalosporins
*bla*_NDM-5_	Subclass 'B1' MBL
*bla*_OXA-1_, *bla*_OXA-534_	Class 'D' ESBL oxacillin hydrolyzing
*bla*_OXA-38_	Class 'D' ESBL carbapenem hydrolyzing
*bla*_TEM-1_, *bla*_TEM-178_	Class 'A' broad spectrum ESBL
*ble*-MBL	Bleomycin-binding protein
*cat *(A1, B3, B8)	Chloramphenicol O-acetyltransferase
*dfr *(A5, A12, A14, A17)	Trimethoprim-resistant dihydrofolate reductase
ereA	Erythromycin esterase
ermB	23SrRNA adenine N methyltransferase
mphA	Macrolide 2' phosphotransferase
sat	Secreted autotransporter toxin
sat2	Multidrug efflux RND transporter periplasmic adaptor subunit
qacE delta1	Quaternary ammonium compound efflux SMR transporter
qnrS1	Qionolone resistance pentapeptide repeat protein
rmtB1	16SrRNA guanine N(7) methyl transferase
*sul1*, *sul2*	Sulfonamide-resistant dihydropteroate synthase
*tet (*A, B, D)	Tetracycline efflux MFS transporter
*16S *(*rrsB*, *rrsC*, *rrsH*)	Spectinomycin, tetracycline, gentamicin, kasugamycin
23S	Macrolide resistance
folP	Carbapenem resistance under development
*gyr *(A, B)	Quinolone resistance
*par *(C, E)	Quinolone resistance
*pmr *(A, B)	Resistance to polymyxin
rpoB	Resistance to rifampicin
ymgB	Biofilm/acid resistance regulator
*ybt *(P, Q)	Yersiniabactin ABC transporter adenosine ATP binding/permease protein
iss	Increased serum survival lipoprotein
ftsI	*Escherichia *aztreonam resistant
espX1	Type III secretion system effector
iutA	Ferric aerobactin receptor
capU	Putative hexosyltransferase
cyaA	*Escherichia *fosmidomycin resistant
*Iro *(D, E)	Catecholate siderophore esterase
glpT	*Escherichia *fosfomycin resistant
iucD	NADPH-dependent L-lysine N(6)-monooxygenase
iucC	NIS family aerobactin synthetase
iucB	N(6)-hydroxylysine O-acetyltransferase
iucA	Aerobactin synthase
iha	Bifunctional siderophore receptor/adhesin
papA	P fimbria major subunit
lpfA	Long polar fimbria major subunit
eilA	HilA family transcriptional regulator
glpT	*Escherichia *fosfomycin resistant
pcoE	Copper/Cu(+) resistance system metallochaperone
pcoS	Copper/Cu(+) resistance membrane-spanning protein
pcoR	Copper/Cu(+) response regulator transcription factor
pcoD	Copper/Cu(+) resistance inner membrane protein
pcoC	Copper/Cu(+) resistance system metallochaperone
pcoB	Copper/Cu(+)-binding protein
pcoA	Multicopper oxidase
silP	Silver/Ag(+)-translocating P-type ATPase
silA	Cu(+)/Ag(+) efflux RND transporter permease subunit
silB	Cu(+)/Ag(+) efflux RND transporter periplasmic adaptor subunit
silF	Cu(+)/Ag(+) efflux RND transporter periplasmic metallochaperone
silC	Cu(+)/Ag(+) efflux RND transporter outer membrane channel
silR	Copper/silver response regulator transcription factor
silS	Copper/silver sensor histidine kinase
silE	Silver-binding protein
lpfA	Long polar fimbria major subunit
senB	Enterotoxin production-related protein
mchB	Microcin H47
mchF	Microcin H47 export transporter peptidase/ATP-binding subunit
sfaF	S/F1C fimbrial biogenesis usher protein
fdeC	Intimin-like adhesin
Vactox	Vacuolating autotransporter toxin
ireA	TonB-dependent siderophore receptor
papC	P fimbrial usher protein
papE	P fimbrial minor subunit
papF	P fimbrial tip protein
papG	P fimbria tip G-adhesin
hlyA-alpha	RTX toxin hemolysin

Conventional antimicrobial susceptibility testing revealed that 15 (50%) isolates were resistant to imipenem, 10 (33.33%) were resistant to aminoglycosides, 13 (43.33%) were resistant to piperacillin-tazobactam, 17 (56.66%) were resistant to cephalosporins, and 14 (46.66%) were resistant to nitrofurantoin. Strain-specific antibiotic resistance genes, virulence genes, and phenotypic antibiotic susceptibility profiles are detailed in Table 2.

**Table 2:** Details of antimicrobial resistance and virulence genes, and phenotypic susceptibility results of the isolates EC: *Escherichia coli*, M: male, F: female, UTI: urinary tract infection, CKD: chronic kidney disease, I: imipenem, AG: aminoglycosides, PTZ: piperacillin-tazobactam, CE: cephalosporins, NIT: nitrofurantoin, S: sensitive, R: resistant

Strain number	Age/sex	Clinical diagnosis	Resistance/virulence genes detected	Phenotype				
				I	AG	PTZ	CE	NIT
EC-249	24/F	Third-trimester pregnancy-UTI	*aph3Ib*,* aph6Id*,* bla*EC-15,* sul2*,* 16SrrsB*,* 23S-123S*,* folP*,* gyr *(*A*,* B*),* par *(*C*,* E*)*, pmr *(*A, B*),* rpoB*	R	S	S	R	S
EC-250	35/M	Renal calculi with UTI	*aac3IIe*, *aadA5*, *bla*_CTX-M-15_, *bla*EC-5, *dfrA17*, *mphA*, *qacEdelta1*, *sul1*, *16SrrsB*, *23S-123S*, *folP*, *gyr *(*A*,* B*) *par *(*C*, *E*), *pmr *(*A*, *B*), *rpoB*	R	S	S	S	R
EC-251	72/M	Urinary calculi	*aac3IId*, *aac6Ib*, *aac6Ibcr5*, *aadA5*, *aph3Ib*, aph6Id, *bla*_CTX-M-15_, *bla*_EC-5_, *bla*_OXA-1_, *bla*_TEM-1_, *catB3*, *catB8*,* dfrA17*, *mphA*, *qacEdelta1*, *sul1*, *sul2*,* tetA*, *16SrrsB*, *23S-123S*, *folP*, *gyr *(*A*, *B*), *par *(*C*, *E*), pmr (*A*, *B*), *rpoB*	S	R	R	S	R
EC-252	26/M	Recurrent UTI	*aph3Ia*, *aph6Id*, *bla*_CTX-M-15_, *bla*EC-18, *bla*_TEM-1_, *tetA*, *16SrrsH*, *23S-123S*, *folP*, *gyr *(*A*, *B*), *par *(*C*, *E*), *pmr *(*A*, *B*), *rpoB*	R	S	S	R	S
EC-254	37/M	CKD	*aac3IIe*, *aac6Ib*, *aac6Ibcr5*, *aph3Ib*, *aph6Id*, *bla*_CTX-M-15_, *bla*EC-5, *bla*_OXA-1_, *bla*_TEM-1_, *catB3*, *dfrA14*, *mphA*, *qacEdelta1*, *sul2*, *tetA*, *16SrrsB*, *16SrrC*, *23S-123S*, *folP*, *gyr *(*A*, *B*), *par *(*C*, *E*), pmr (*A*, *B*), *rpoB*	R	S	S	R	R
EC-255	49/F	Renal calculi with UTI	*aadA5*, *bla*_CTX-M-15_, *bla*EC-5, *dfrA17*, *mphA*, *qacEdelta1*, *sul1*, *tetA*, *16SrrsB*, *23S-123S*, *folP*, *gyr *(*A*, *B*), *par *(*C*, *E*), *pmr *(A, B), *rpoB*	R	S	S	R	R
EC-256	45/M	UTI	*aac3IId*, *aac6Ib*, *aac6Ibcr7*, *aadA5*, *aph3Ib*, *aph6Id*, *bla*EC-16, *catA1*, *catB8*, *dfrA17*, mphA, *qacEdelta1*, *sul1*, *tetB*, *16SrrsC*, *23S-123S*, *folP*, *gyr *(*A*, *B*), *par *(*C*, *E*), *pmr *(*A*, *B*), *rpoB*	S	S	S	S	S
EC-258	26/F	Third-trimester pregnancy-UTI	*aac6 30*, *aac6Ib*, *aac6Ibcr5*, *aadA5*, *arr3*, *bla*_CMY-42_,* bla*EC-19, *bla*_OXA-1_, *catA1*, *catB3*, *dfrA17*, *qacEdelta1*, *sul1*, *tetB*, *16SrrsH*, *23S-123S*, *folP*, *gyr *(*A*, *B*), *par *(*C*, *E*), *pmr *(*A*, *B*), *rpoB*	S	S	S	S	S
EC-259	68/M	Recurrent UTI	*aac3IIe*, *bla*_CTX-M-15_, *bla*EC-5, *mphA*, *16SrrsB*, *23S-123S*, *folP*, *gyr *(*A*, *B*), *par *(*C*, *E*), *pmr *(*A*, *B*), *rpoB*	R	S	S	S	R
EC-260	21/F	Third-trimester pregnancy-UTI	*aadA5*, *bla*_CMY-2_, *bla*EC-5, *dfrA17*, *qacEdelta1*, *sul1*, *16SrrsB*, *23S-123S*, *folP*, *gyr *(*A*, *B*), *par *(*C*, *E*), *pmr *(*A*, *B*), *rpoB*	S	S	S	S	S
EC-261	70/M	UTI	*aph3Ib*, *aph6Id*, *bla*_CMY-42_, *bla*EC-15, *bla*_OXA-181_, *ereA*, *ermB*, *mphA*, qnrS1, *sul2*, *tetB*, *16SrrsB*, *23S-123S*, *folP*, *gyr *(*A*, *B*), *par *(*C*, *E*), *pmr *(*A*, *B*), *rpoB*	R	S	R	S	S
EC-262	35/F	Renal calculi with UTI	*aac3IIe*, *aac30*, *aac6Ib*, aac6Ibcr5, *aadA5*, *bla*_CTX-M-15_, *bla*EC-19, *bla*_OXA-1_, *catA1*, *catB3*, *dfrA17*, *mphA*, *qacEdelta1*, *sul1*, *tet *(*A*, *B*), *16SrrsH*, *23S-123S*, *folP*, *gyr *(*A*, *B*), *par *(*C*, *E*), *pmr *(*A*, *B*), *rpoB*	R	S	R	S	R
EC-263	70/M	UTI	*aac3IIe*, *aac30*, *aac6Ib*, *aac6Ibcr5*, *bla*_CMY-42_, *bla*_CTX-M-15_, *bla*EC-8, *bla*_OXA-1_, *bla*_TEM-178_, *catB3*, *qnrS1*, *tetB*, *16SrrsH*, *23S-123S*, *folP*, *gyr *(*A*, *B*), *par *(C, *E*), *pmr *(*A*, *B*), *rpoB*	R	R	R	R	S
EC-266	45/F	UTI	*bla*EC-5, *16SrrsB*, *23S-123S*, *folP*, *gyr *(*A*, *B*), *par *(*C*, *E*), *pmr *(*A*, *B*), *rpoB*	R	S	R	R	S
EC-1216	69/M	UTI	*aac630*, *aac6Ib*, *aac6Ibcr5*, *aadA2*, *bla*_AFM-1_, *bla*_CTX-M-15_, *bla*EC-8, *bla*_OXA-1_, *bla*_TEM-1_, *ble*, *catB3*, *dfrA12*, *mphA*, *qacEdelta1*, *rmtB1*, *sul1*, *16SrrsC*, *23S-123S*, *folP*, *gyr *(*A*, *B*), *par *(*C*, *E*), *pmr *(*A*, *B*), *rpoB*	R	R	R	R	S
EC-1217	70/M	Acute intraperitoneal obstruction-UTI	*aac630*, *aac6Ib*, *aac6Ibcr5*, *aadA5*, *aph6Ic*, *bla*_CMY-42_, *bla*_CTX-M-15_, *bla*EC-15, *bla*_OXA-534_, *catB3*, *catB8*, *dfrA17*, *mphA*, *qacEdelta1*, *sul1*, *tetA*, *16SrrsH*, *23S-123S*, *folP*, *gyr *(*A*, *B*), *par *(*C*, *E*), *pmr *(*A*, *B*), *rpoB*	R	R	R	R	R
EC-1218	17/M	UTI	*aac630*, *aac6Ib*, *aac6Ibcr5*, *aadA2*, *bla*_AFM-1_, *bla*_CTX-M-15_, *bla*EC-8, *bla*_OXA-1_, *bla*_NDM-5_, *bla*_TEM-1_, *ble*, *catB3*, *dfrA12*, *qacEdelta1*, *rmtB1*, *sul1*, *16SrrsH*, *23S-123S*, *folP*, *gyr *(*A*, *B*), *par *(*C*, *E*), *pmr *(*A*, *B*), *rpoB*	R	R	R	R	S
EC-1219	40/M	UTI	*aac630*, *aac6Ib*, *aac6Ibcr5*, *aadA2*, *bla*_CTX-M-15_,* bla*EC-8, *bla*_OXA-1_, *bla*_NDM-5_, *bla*_TEM-1_, *ble*, *catB3*, *dfrA12*, *qacEdelta1*, *rmtB1*, *sul1*, *16SrrsC*, *23S-123S*, *folP*, gyr (*A*, *B*), *par *(*C*, *E*), *pmr *(*A*, *B*), *rpoB*	S	S	S	S	S
EC-1221	66/M	UTI	*bla*_CTX-M-27_, *bla*EC-5, *16SrrsB*, *23S-123S*, *folP*, *gyr *(*A*, B), *par *(*C*, *E*), *pmr *(*A*, *B*), *rpoB*	R	S	R	R	S
EC-1244	65/M	UTI	*aac3IIe*, *aac630*, *aac6Ib*, *aac6Ibcr5*, *aadA1*, *aadA13*, *aph3Ib*, *aph6Id*,* bla*_CTX-M-15_, *bla*EC-8, *bla*_OXA-1_, *bla*_TEM-1_, *catA1*, *catB3*, *dfrA1*, *ermB*, *mphA*, *sul2*, *sat2*, *tetD*, *16SrrsH*, *23S-123S*, *folP*, gyr (*A*, *B*), par (*C*, *E*), *pmr *(*A*, *B*), *rpoB*	S	S	S	R	R
EC-1756	72/M	UTI	*parE_S458A*, *parC_S80I*, blaEC, *iss*, *fdeC*, *mdtM*, *gyrA_D87N*, *gyrA_S83L*, *ymgB*, *papG-II*, *papF*, *papC*, *papA*, *ybtQ*, *ybtP*, *bla*_CMY-42_, *espX1*, *ftsI_N337NYRIN*, *astA*, capU, *bla*_NDM-5_, *ble*, *sul1*, *qacEdelta1*, *aadA2*, *dfrA12*, *acrF*, *mphA*, *bla*_TEM-1_, *rmtB1*, *ermB*	S	R	R	R	R
EC-1757	19/M	CKD with hydronephrosis	*aadA2*, *acrF*, *bla*_CMY-14_, *bla*EC, *dfrA12*, *ermB*, *ftsI_N337NYRIN*, *gyrA*, *mdtM*, *mphA*, *par *(*C*, *E*), *sul1*, *tetB*, *ymgB*, *ybt *(*P*, *Q*), *fdeC*, *iss*, *espX1*, *qacEdelta1*, *iutA*, *capU*	S	S	S	R	R
EC-1758	48/M	Septic shock-UTI	*aac3IIe*, *aac6Ibcr5*, *aadA2*, *acrF*, *bla*_CTX-M-15_, *bla*EC, *bla*_OXA_, bla_TEM-1_,*cyaA_S352T*, *catB3*, *dfrA12*, *ermB*, *emrD*, *glpT_E448K*, *gyrA*, *mdtM*, *mphA*, *sul1*, *par *(*C*, *E*), *fdeC*, *iroE*, *iroD*, *espX1*, *sat*, *iutA*, *iuc *(*A*-*D*), *qacEdelta1*, *iha*, *papA*, *capU*, *ipfA*, *eilA*, *ymgB*	S	R	R	R	R
EC-1759	67/M	UTI	*aac3IId*, aac6Ibcr5, *aadA5*, *acrF*, *aph3Ib*, *aph6Id*, *arr*, *bla*_CMY-4_,* bla*EC, *bla*_OXA-1_, *bla*_TEM-1_, *catB3*, *dfrA17*, *ftsI_N337NYRIN*, *glpT_E448K*, *nsfA-R203C*, *glpT_E448K*, *gyrA*, *mdtM*, *mphA*, *sul *(*1*, *2*), *par *(*C*, *E*), *tetB*, *espX1*, *fdeC*, *ybtP-Q*, *pco *(*A*- *E*, *R*, *S*), sil (*A*-*C*, *E*, *F*, *R*, *S*), *ymgB*,*qacEdelta1*, *IpfA*	S	S	R	S	S
EC-1760	27/F	UTI	*acrF*, *bla*EC, *emr *(*D*, *E*), *glpT_E448K*, *gyrA*, *mdtM*, *pmrB*, *iss*, *ybt *(*P*, *Q*), *iuc *(*A*-*D*), *iutA*, *senB*, *mch *(*B*, *F*), *sfaF*, *fdeC*, *vactox*, iro (*B*-*E*, *N*), ireA, *pap *(*C*, *E*, *F*), *papG-II*, *hlyA-alpha*, *glpT_E448K*, *capU*, *iha*, *ymgB*	S	S	S	S	S
EC-1761	27/F	UTI	*aad *(*A2*, *A5*), *acrF*, *aph3Ib*, *aph6Id*, *bla*_CTX-M-27_, *bla*EC, *dfrA17*, *emrD*, *glpT_E448K*, *gyrA*, *mdtM*, *mphA*, *ptsl_V25I*, *sul *(*1*, *2*), *par *(*C*, *E*), *pmrB*, *tetA*, *uhpT_E350Q*, *afaC*, *nfaE*, *fdeC*, *ybt *(*P*, *Q*), *emrE*, *ymgB*, *sat*, *iutA*, *iuc *(*A*-*D*), *iha*, *papA*, *emrD*	S	S	S	R	S
EC-1763	65/M	UTI	*acrF*, *bla*_CTX-M-15_, *bla*EC, *emr *(*D*, *E*), *glpT_E448K*, *gyrA*, *mdtM*, *pmrB*, *pmrB_E123D*, *fdeC*, *vactox*, *emrE*, *gyrA_S83L*, *glpT_E448K*, *senB*, *iro *(*B*-*E*), *iroN*, *focG*, *sfaF*, *mchF*, *mchB*, *iutA*, *iuc *(*A*-*D*), *hlyA*-*alpha*, *acrF*, *iha*, *ireA*, *ymgB*, *papH*, *papC*, *papE*, *papF*, *cnf1*, *ybt *(*P*-*Q*), *papG-II, III*, *iss*, *papA*	S	S	S	R	S
EC-1724	33/F	UTI	*aadA2*, *acrF*, *rmtB1*, *bla*_CTX-M-15_, *bla*_CMY-42_, *bla*_NDM-5_, *bla*EC, *bla*_TEM-1_, *ftsI_N337NYRIN*, *ble*, *pmrB_Y358N*, *mdtM*, *glpT_E448K*, *mphA*, *gyrA_D87N*,*gyrA_S83L*, *parC_S80I*, *parE_S458A*, *sul1*, *tet *(*A*, *D*), *dfrA12*	S	S	S	S	R
EC-1765	24/F	UTI	*acrF*, *pmrB_E123D*, *bla*EC, *iss*, *fdeC*, *vactox*, *gyrA_S83L*, *glpT_E448K*, *iro *(*B*-*E, N*), *sfaF*, *mchF*, *mchB*, *ireA*, *ybt *(*P*, *Q*), *emrE*, *iut *(*A*, *D*), *iuc *(*A*-*C*). *hlyA-alpha*, *senB*, *iha*, *papH*, *papC*, *papF*, *papG-II*, *ymgB*, *papA*, *emrD*	S	S	S	S	R
EC-1768	77/M	UTI	*Iss*, *fdeC*, *lpfA*, *uhpT_E350Q*, *mdtM*, *iro *(*B*-*E*,* N*), *iss*, *acrF*, *parE_S458A*, *parC_S80I*, *sul3*, *qacL*, *aadA1*, *cmlA1*, *aadA2*, *dfrA12*, *gyrA_D87N*, *gyrA_S83L*, *glpT_E448K*, *qnrS13*, *bla*_CTX-M-15_, *ymgB*, *mphA*, *tetA*, *espX1*, *bla*EC, *pmrB_Y358N*	R	R	R	R	R

Among the isolates, 26 (86.66%) had revealed the presence of multiple antibiotic-resistant genes with evidence of at least one gene coding for beta-lactamase resistance. The genes identified in this study were *bla*_CTXM-15_ (19/30, 63.33%), *bla*_CTXM-27_ (2/30, 6.66%), *bla*_CMY-2_ (1/30, 3.33%), *bla*_CMY-4_ (1/30, 3.33%), *bla*_CMY-42_ (6/30, 20%), *bla*_CMY-145_ (1/30, 3.33%), *bla*_^OXA-1^_ (11/30, 36.66%), *bla*_OXA-534_ (1/30, 3.33%), *bla*_OXA-181_ (1/30, 3.33%), *bla*_AFM-11_ (2/30, 6.66%), *bla*_TEM-1_ (10/30, 33.33%), *bla*_TEM-178_ (1/30, 3.33%), and *bla*_NDM-5_ (3/30, 10%). Serotyping of the isolates showed the presence of serotype O25 (6, 20%), which was the predominant, followed by serotypes O102 (4, 13.33%), O4 (4, 13.33%), O101 (2, 6.66%), O1 (2, 6.66%), O8 (2, 6.66%), O89 (2, 6.66%), O54 (1, 3.33%), O188 (1, 3.33%), O75 (1, 3.33%), O11 (1, 3.33%), O153 (1, 3.33%), O100 (1, 3.33%), O9 (1, 3.33%), and O16 (1, 3.33%). Among the isolates, a majority belonged to serotype H4 (07, 23.33%) and H6 (7, 23.33%), followed by H9 (3, 10%), H5 (3, 10%), H21 (3, 10%), and H1 (3, 10%). Other H serotypes identified included H28 (1, 3.33%), H30 (1, 3.33%), H18 (1, 3.33%), and H23 (1, 3.33%). The *E*. *coli *serotype found predominantly belonged to O25:H4 (5, 16.66%), followed by O102:H6 (4, 13.33%), O4:H1 (3, 10%), O89:H9 (2, 6.66%), O1:H6 (2, 6.66%), O8:H21 (2, 6.66%), O101:H9 (1, 3.33%), O54:H28 (1, 3.33%), O199:H4 (1, 3.33%), O75:H5 (1, 3.33%), O101:H21 (1, 3.33%), O11:H30 (1, 3.33%), O16:H5 (1, 3.33%), O4:H5 (1, 3.33%), and O9:H23 (1, 3.33%) as shown in Table 3.

**Table 3:** Serotypes of the E. coli strains EC: *Escherichia coli*, *E. coli*: *Escherichia coli*

Strain	Serotype	
	O (cell wall/somatic antigen)	H (flagellar antigen)
EC-249	O101	H9
EC-250	O25	H4
EC-251	O25	H4
EC-252	O54	H28
EC-254	O25	H4
EC-255	O25	H4
EC-256	O188	H4
EC-258	O1	H6
EC-259	O25	H4
EC-260	O75	H5
EC-261	O101	H21
EC-262	O1	H6
EC-263	O102	H6
EC-266	O4	H1
EC-1216	O102	H6
EC-1217	O8	H21
EC-1218	O102	H6
EC-1219	O102	H6
EC-1221	O25	H4
EC-1244	O11	H30
EC-1756	O89	H9
EC-1757	O89	H9
EC-1758	O153	H6
EC-1759	O8	H21
EC-1760	O4	H1
EC-1761	O16	H5
EC-1763	O4	H5
EC-1724	O100	H18
EC-1765	O4	H1
EC-1768	O9	H23

The functions of the housekeeping genes based on which the MLST was carried out and the plasmid replicons identified among the isolates are shown in Table 4.

**Table 4:** Resistance genes and plasmid replicons along with their functions Col: colicinogenic plasmid, TCA: tricarboxylic acid cycle, DNA: deoxyribonucleic acid

Virulence gene/housekeeping gene	Function	Usefulness
adk	Adenylate kinase: catalyzes conversion between adenylate nucleotides	Required for growth and survival
fumC	Fumarase: oxidative TCA cycle enzyme	Probably causes fitness defects in the bladder and kidneys, facilitating UTI
icd	Isocitrate dehydrogenase	Essential for cell growth and energy production
mdh	Malate dehydrogenase	Adaptation of bacteria to the environment (aerobic and anaerobic) and cell growth
purA	Adenylosuccinate synthetase	Invasive properties
recA	DNA recombination/repair protein	Protects against oxidative damage in host cells
*IncB*, *IncFIA*, *IncFIB*, *IncFII*, *IncB*/*O*/*K*/*Z*, IncY, *p0001*	Incompatibility (*Inc*) group	Carry drug resistance genes
*Col156*, *col*(*MG828*), *col8282*, *colBS512*, *colRNAI*	*Col*-like plasmid replicons	Carry drug resistance genes

A total of 16 MLST variants were identified among the analyzed samples. Of the MLST types identified, ST-131 (7, 23.33%) was the predominant one, followed by ST-167 (3, 10%), ST-12 (3. 10%), ST-5954 (3, 10%), ST-648 (2, 6.66%), ST-410 (2, 6.66%), ST-156 (1, 3.33%), ST-448 (1, 3.33%), ST-14 (1, 3.33%), ST-1284 (1, 3.33%), ST-405 (1, 3.33%), ST-38 (1, 3.33%), ST-8881 (1, 3.33%), ST-2851, ST-827 (1, 3.33%), and ST-2006 (1, 3.33%) as shown in Table 5.

**Table 5:** Sequence types and plasmid replicons identified among the isolates EC: *Escherichia coli*, MLST: multilocus sequence typing, ST: sequence type, *Inc*: incompatibility group, *Col*: colicinogenic plasmid

Strain	MLST type	Housekeeping genes	Plasmid replicons
EC-249	ST-167	*adk*, *fumC*, *gyrB*, *icd*, *mdh*, *purA*, *recA*	IncB
EC-250	ST-131	*adk*, *fumC*, *gyrB*, *icd*, *mdh*, *purA*, *recA*	*IncFIA*, *IncFIB*, *IncFII*
EC-251	ST-131	*adk*, *fumC*, *gyrB*, *icd*, *mdh*, *purA*, *recA*	*Col156*, *IncFIB*, *IncFII*
EC-252	ST-156	*adk*, *fumC*, *gyrB*, icd, *mdh*, *purA*, *recA*	*IncFIB*, *IncFII*
EC-254	ST-131	*adk*, fumC, *gyrB*, *icd*, *mdh*, *purA*, *recA*	*IncFIA*, *IncFIB*, *IncFII*
EC-255	ST-131	*adk*, *fumC*, *gyrB*, *icd*, *mdh*, *purA*, *recA*	*Col156*, *IncFIA*, *IncFIB*, *IncFII*
EC-256	ST-448	*adk*, *fumC*, *gyrB*, *icd*, *mdh*, *purA*, *recA*	*ColBS512*, *IncFIA*, *IncFIB*, *IncFII*
EC-258	ST-648	*adk*, *fumC*, *gyrB*, *icd*, *mdh*, *purA*, *recA*	*ColBS512*, *ColMG828*, IncFIA, *IncFIB*, *IncFII*, *IncI1*
EC-259	ST-131	*adk*, *fumC*, *gyrB*, *icd*, *mdh*, *purA*, *recA*	*IncFIA*, *IncFIB*, *IncFII*
EC-260	Single locus variant of ST-14	*adk*, *fumC*, *gyrB*, *icd*, *mdh*, *purA*, *recA*	*Col156*, *ColRNAI*, *ColBS512*, *IncFIA*, *IncFIB*, *IncFII*, *IncI1*
EC-261	ST-1284	*adk*, *fumC*, *gyrB*, *icd*, *mdh*, *purA*, *recA*	*ColKP3*, *ColRNAI*, *ColBS512*, *ColpVC*, *IncFIA*, *IncFIB*, *IncFII*, *IncI1*, *IncX3*
EC-262	ST-648	*adk*, *fumC*, *gyrB*, *icd*, *mdh*, *purA*, *recA*	*Col8282*, *IncFIA*, *IncFIB*, *IncFII*
EC-263	ST-405	*adk*, *fumC*, *gyrB*, *icd*, *mdh*, *purA*, *recA*	*ColRNAI*, *ColBS512*, *ColMG828*, *IncFIA*, *IncFIB*, *IncFII*, *IncI1*, *IncX1*
EC-266	ST-12	*adk*, *fumC*, *gyrB*, *icd*, *mdh*, *purA*, *recA*	*IncFIB*, *IncFII*
EC-1216	ST-5954	*adk*, *fumC*, *gyrB*, *icd*, *mdh*, *purA*, *recA*	*ColMG828*, *IncFIA*, *IncFII*, *p0111*
EC-1217	ST-410	*adk*, *fumC*, *gyrB*, *icd*, *mdh*, *purA*, *recA*	*Col156*, *ColRNAI*, *ColBS512*, *IncFIA*, *IncFIB*, *IncFII*, *IncI1*
EC-1218	ST-5954	*adk*, *fumC*, *gyrB*, *icd*, *mdh*, *purA*, *recA*	*ColBS512*, *ColMG828*, *IncFIA*, *IncFII*, *p0111*
EC-1219	ST-5954	*adk*, *fumC*, *gyrB*, *icd*, *mdh*, *purA*, *recA*	*ColMG828*, *IncFIA*, *IncFII*, *p0111*
EC-1221	ST-131	*adk*, *fumC*, *gyrB*, *icd*, *mdh*, *purA*, *recA*	*Col156*, *ColRNAI*, *IncFIA*, *IncFIB*, *IncFII*
EC-1244	ST-38	*adk*, *fumC*, *gyrB*, *icd*, *mdh*, *purA*, *recA*	*IncFIB*, *IncFII*, *IncX4*
EC-1756	ST-167	*adk*, *fumC*, *gyrB*, *icd*, *mdh*, *purA*, *recA*	Col*(BS512)*, *Col440I*, *IncFIA*, *IncFIB*, *IncFII*, *IncI (Gamma)*, *IncX4*
EC-1757	ST-167	*adk*, *fumC*, *gyrB*, *icd*, *mdh*, *purA*, *recA*	*IncFIA*, *IncFIB (AP001918)*, *IncFII*, *IncI (Gamma)*
EC-1758	ST-8881	*adk*, *fumC*, *gyrB*, *icd*, *mdh*, *purA*, *recA*	*IncFIA*, *IncFIB (AP001918)*, *IncFII (pRSB107)*
EC-1759	ST-410	*adk*, *fumC*, *gyrB*, *icd*, *mdh*, *purA*, *recA*	*IncB*/*O*/*K*/*Z*, *IncFIA*, *IncFIB (AP001918)*, *IncFII (pAMA1167-NDM-5)*,* IncI (Gamma)*
EC-1760	ST-12	*adk*, *fumC*, *gyrB*, *icd*, mdh, *purA*, *recA*	*IncFIB (AP001918)*, *IncFII*
EC-1761	ST-131	adk, fumC, gyrB, icd, mdh, purA, recA	*IncFIA*, *IncFIB*(AP001918), *IncFII*(pRSB107)
EC-1763	ST-12	adk, fumC, gyrB, icd, mdh, purA, recA	*IncFIB*(AP001918), *IncFII*
EC-1724	ST-2851	adk, fumC, gyrB, icd, mdh, purA, recA	*Col *(MG828), *Col *(pHAD28), *IncF1A*, *IncF1B *(pNDM-Mar), *IncFII*, *IncHI1B *(pNDM-MAR), *IncI *(Gamma)
EC-1765	ST-827	adk, fumC, gyrB, icd, mdh, purA, recA	*IncB*/*O*/*K*/*Z*, *IncFIB*(AP001918), *IncFII*
EC-1768	2006	adk, fumC, gyrB, icd, mdh, purA, recA	*Col156*, *ColRNAI*, *IncFII*(pCoo), *IncFIB *(AP001918), *IncI*(Gamma), *IncY*

## Discussion

Among the several public health concerns encountered globally, resistance of microbes to antimicrobial agents has become a tough nut to crack. AMR could be attributed to factors such as irrational and indiscriminate use of antimicrobial agents and lack of newer and more efficient drugs being developed by pharmaceutical industries owing to the high cost associated with drug development. Antibiotic use began with penicillin during the Second World War (1940). However, slowly and gradually, bacterial species resistant to penicillin emerged. This led to using newer penicillins such as methicillin and other antibiotics such as tetracyclines, erythromycin, and aminoglycosides such as gentamicin. Later, cephalosporins, including ceftazidime, were prescribed to treat bacterial infections. However, due to irresponsible prescription practices, bacterial species resistant to most of antimicrobial agents started to emerge, and such bacterial species were labeled as MDR, pan-drug-resistant (PDR), and extensively drug-resistant (XDR) bacteria [15].

MDR strains are identified based on the resistance shown by bacteria to at least one drug under three or more antimicrobial agent categories. When a bacterium is found resistant to at least one antimicrobial agent in most categories, it is identified as an XDR bacterium. A bacterial species showing resistance to all the antibiotics under different categories is labeled PDR bacteria [16].

Increased AMR has resulted in the rise of morbidity and mortality among affected patients. Additionally, AMR could cause an economic burden on patients attributed to extended hospital stays and prolonged antibiotic use [17].

Beta-lactamase resistance genes

Enterobacteriaceae members, including *E*. *coli*, are noted to develop intrinsic resistance to antibiotics and transferable resistance (chromosomes and plasmids). This forces us to monitor the presence of AMR genes among bacterial isolates. Intrinsic resistance was attributed to AmpC-beta-lactamase and broad-spectrum beta-lactamases such as *bla*_TEM-1_, *bla*_TEM-2_,* bla*_SHV-1_, and *bla*_OXA-1_ [18].

The beta-lactam group of antibiotics are those antibiotics that interfere with cell wall synthesis. Penicillin, ampicillin, and amoxicillin are a few examples of antibiotics that possess a beta-lactam ring. However, bacterial species have been able to counter the activities of beta-lactam antibiotics by producing beta-lactamase enzymes that catalyze beta-lactam antibiotics, thereby inactivating them. Considering this, scientists have developed beta-lactamase inhibitors that could be combined with beta-lactam antibiotics, which enables them to counter the activities of beta-lactamases produced by bacteria. Amoxycillin is a beta-lactam antibiotic when combined with beta-lactamase inhibitors such as clavulanic acid to counter the beta-lactamases produced by bacteria.

Over time, bacteria have acquired the ability to survive the action of beta-lactam antibiotics. Infections due to these bacteria were treated by antibiotics such as cephalosporins. Further, a few bacterial species started showing resistance to the penicillin group and the narrow and broad-spectrum cephalosporin group of antibiotics. Such bacteria were labeled as extended-spectrum beta-lactamase (ESBL)-producing bacterial species. It was identified that bacteria develop resistance to lower cephalosporins such as cefazolin and cephalothin consisting of genes such as Temoniera-1 (TEM-1) and TEM-2. Following the availability of higher cephalosporins such as cefotaxime, ceftriaxone, and ceftazidime, among others, with broad-spectrum activities, bacteria with TEM-1 and TEM-2 were inhibited. Later, bacteria with lowered susceptibility to broad-spectrum cephalosporins with oxyimino side chain started to appear, which were found to possess the sulfhydryl variable (SHV) gene [19].

Beta-lactamases were classified into four types (A, B, C, and D), wherein class A, C, and D were identified as serine beta-lactamases and class B was labeled as metallo-beta-lactamases. More ESBLs have been identified in bacteria, which included AmpC, hydrolysis of cefotaxime Munich (CTX-M), oxacillin hydrolyzing type (OXA), complex mutant derived from TEM (CMT), inhibitor-resistant TEM (IRT), *Pseudomonas *extended resistance (PER), Vietnamese ESBL (VEB), Guiana extended spectrum (GES), Belgium ESBL (BEL), *Serratia fonticola *(SFO), *Klebsiella oxytoca *(OXY), and others [20].

Among the different classes of beta-lactamases, class A (Serratia marcescens enzyme (SME), imipenem-hydrolyzing beta-lactamase (IMI), not metalloenzyme carbapenemase (NMC), GES, and *Klebsiella pneumoniae* carbapenemase (KPC) families), B (active on imipenem (IMP), Verona integron-encoded metallo-beta-lactamase (VIM), and New Delhi metallo-beta-lactamase (NDM)), and D (OXA) are known to develop resistance to penicillin, cephalosporins, monobactams, and carbapenem group antibiotics. Class B beta-lactamases known as metallo-beta-lactamases have a zinc moiety [21-23].

A high prevalence of *bla*_CTX-M_ (19, 63.33%) type ESBL was witnessed in the current study. A similar finding was reported from Bangladesh wherein *E*. *coli *isolated in extraintestinal specimens demonstrated *bla*_CTX-M_ prevalence of 52% [24]. The study noticed a lower prevalence of *bla*_TEM_ (20%) and *bla*_OXA-1_ (17%) compared to the present study results, wherein *bla*_TEM_ and* bla*_OXA-1_ showed a higher prevalence of 36.66% (11/30). Virulence determinants such as iutA that define extraintestinal pathogenic *E*. *coli *(ExPEC) were demonstrated in a high (62%) number of strains compared to our study (5, 16.66%) [24].

Results of genomic analysis showed *bla*_TEM-1_ (100%), *bla*_CTX-M-15_ (16%), and *bla*_CMY -42_ (3%) in the *E*. *coli *isolated from river water in Delhi, India [25]. In the present study, we found *bla*_TEM-1_ (11, 36.66%), *bla*_CTX-M-15_ (19, 63.33%), and *bla*_CMY-42_ (6, 20%). Most isolates produced *bla*_CTX-M_ ESBL, and many belonged to ST-131, as noticed in the present study. Three (10%) isolates showed the presence of *bla*_NDM-5_ in the present study. In previous studies from China that evaluated carbapenem-resistant *E*. *coli *strains, 64% revealed the presence of the *bla*_NDM-5_ gene, and a predominance of *bla*_CTX-M-15_-carrying ST-131 was noticed [26,27].

The most common STs identified in our study were ST-131 (7/30), followed by ST-167 (3/30), ST-12 (3/30), and ST-5954 (3/30). Results from a previous Indian study showed a predominance of ST-167, followed by ST405 and ST410 [28].

ST-131 (O25:H4)

ST-131 clones were first identified in 2003. However, they became highly prevalent strains of ExPEC by the year 2008. These *E*. *coli *strains have become prominent due to their ability to carry the *bla*_CTX-M-15_ gene and demonstrate ESBL activities. Among extraintestinal infections, *E*. *coli *li is the primary cause of UTIs. It was also observed that most ST-131 *E*. *coli *demonstrate the same serotype (O25:H4). This finding was also confirmed by the results of the current study, wherein 85.71% (6 out of 7) of the ST-131 *E*. *coli *showed an O25:H4 serotype. There was one ST-131 that revealed the O16:H5 serotype, which was first identified in Japan in 2012 and later in other countries [29].

The ST-131 cone was also identified as being responsible for recurrent UTIs. Moreover, ST-131 was frequently associated with hospital- and community-acquired infections. Colonization in animals and birds and MDR were a few characteristic features established in ST-131 clones [30].

ST-167 (O89:H9-2, O101:H9-1)

The first report of *bla*_NDM-1_ carriage among ST-167 strains was noticed in China. The ST-167 was found to carry *bla*_NDM-5_ genes among human and dog isolates [31-33]. These reports suggest the potential for ST-167 to carry antimicrobial resistance genes that contribute to MDR. Additionally, a recent study performed WGS and identified the conjugative plasmid present in ST-167 that carried *bla*_NDM-5_ [34].

*Escherichia coli *ST-167 was identified as a predominant clone that carried carbapenem resistance through conjugative plasmids (*IncFII *and *IncX3*, *IS26*, and *Tn3*) that can potentially contribute to horizontal gene transfer [35]. More numbers (12/50, 24%) of *E*. *coli *ST-167 strains acquired from hospitalized patients, mostly isolated from urine, were noted to carry *bla*_NDM_ genes on conjugative plasmids (*IncF*, *IncX*, and *IncH*) [36].

ST-12 (O4:H1-two, O4:H5-one)

In the current study, this ST was found to carry several resistance and virulence genes such as *acrF*, *bla*_CTX-M-15_, *bla*EC, *emr *(*D*, *E*), *glpT_E448K*, *mdtM*, *iss*, *ybt *(*P*, *Q*), *iuc *(*A*-*D*), *iutA*, *senB*, *much *(*B*, *F*), *sfaF*, *fdeC*, *vactox*, *iro *(*B*-*E*, *N*), *ireA*, *pap *(*C*, *E*, *F*), *papG-II*, *hlyA-alpha*, *glpT_E448K*, *capU*, *iha*, *ymgB*, *bla*EC-5, *16SrrsB*, *23S-123S*, *folP*, *gyr *(*A*, *B*), *par *(*C*, *E*), *pmr *(*A*, *B*), and *rpoB*, as well as plasmids such as *IncFII* and *IncFIB (AP001918)*. This ST was among the dominant types isolated from dogs in Spain. Among the virulence genes identified, several were identified in the present study, such as *iutA*, *sat*, *iss*, and *papC *[37].

ST-410 (O8:H21)

This strain type was previously described as a high-risk variant that can develop antimicrobial resistance and high virulence capabilities such as *E*. *coli *ST-131. In this study, two isolates belonging to ST-410 (2/30, 6.66%) were recognized. Further, both these STs belonged to the O8:H21 serotype [38].

MLST of *E*. *coli *has been a familiar ST known to carry drug resistance genes that make it MDR. These strains can harbor the resistance genes and potentially transmit the drug resistance genes, intraspecies and interspecies. This is evident from a recent report that demonstrated the transfer of the *bla*_KPC-2_ gene from *E*. *coli *ST-410 to *Klebsiella pneumoniae *through *IncX3 *plasmids [39].

ST-648 (O1:H6)

In the current study, this ST was noted to carry *bla*_CMY-42_, *bla*EC-19, bla_OXA-1_, and *bla*_CTX-M-15_. This ST was previously identified in birds, dogs, cats, and horses [40,41]. ST-648 strain with O1:H6 serotype was also identified in UTI-causing *E*. *coli *isolates from Brazil [42]. This ST was observed among *E*. *coli *isolates from wastewater pumps of the community. Similar to the results of this study, plasmid *Col8282 *and *bla*_CTX-M-15_ resistance genes were observed [43]. ST-648 was the second most common ST identified with an O1:H6 serotype (5.6%) combination, second to ST-131 [44]. ST-648 was among the predominant STs noted from *E*. *coli *isolated from human feces, sewage, and foodstuffs in England. Most of these isolates were noted to carry *bla*_CTX-M-15_ [45].

It was confirmed that ST-648 does not possess any specific host affliction. However, ST-648 was frequently associated with MDR and high virulence capabilities, including biofilm formation with the potential to cause invasive infections such as bacteremia [46].

ST-405 (O102:H6)

In the present study, only one ST-405 was identified. Additionally, this ST carried several resistance and virulence genes such as *aac3IIe*, *aac30*, *aac6Ib*, *aac6Ibcr5*, *bla*_CMY-42_, *bla*_CTX-M-15_, *bla*EC-8, *bla*_OXA-1_, *bla*_TEM-178_, *catB3*, *qnrS1*, *tetB*, *16SrrsH*, *23S-123S*, *folP*, *gyr *(*A*, *B*), *par *(*C*, *E*), *pmr *(*A*, *B*), and *rpoB*. It was observed in a previous study that this ST was highly pathogenic and was a frequent cause of urosepsis. Urosepsis is an invasion of bacteria causing UTI into the blood, resulting in bacteremia. Additionally, this strain was noted to carry resistance genes, including *bla*_CTX-M-15_, enabling them to become MDR [47].

This MLST with the same combination of serotype (O102:H6) was isolated from a patient suffering from septicemia in Mozambique. Moreover, WGS revealed that this strain carried *bla*_NDM_ gene alongside *bla*_CTX-M-15_, *bla*_TEM-1_, *aadA2*, *sul1*, *gyrA*, *parC*, *parE*, and *dfrA12 *genes [48]. A previous study identified plasmid replicons (*IncFIB*, *IncFII*, *IncFIA*, *IncI1*, *IncX1*, *IncFIC*, and *Col*) that could carry resistance and virulence genes in ST-405 [49].

In the present study, ST-405 was found to carry plasmid replicons, including *ColRNAI*, *ColBS512*, *ColMG828*, *IncFIA*, *IncFIB*, *IncFII*, *IncI1*, and *IncX1*.

Other STs identified in the present study that are scarcely reported in the literature

ST-5954 (O102:H6)

Two out of the three strains (66.66%) from this ST were noted to carry *bla_NDM-5_* genes. This ST accounted for 10% (3/30) of the strains in the present study. This ST revealed the presence of plasmid genes such as *ColMG828*, *ColBS512*, *ColMG828*, *IncFIA*, *IncFII*, and *p0111*. Among the genes responsible for ESBL, this isolate showed the presence of *bla*_AFM-1_, *bla*_CTX-M-15_, *bla*_EC-8_, *bla*_OXA-1_, *bla*_TEM-1_, and *bla*_NDM-5_ genes, among others.

ST-1284 (O101:H21)

In the present study, this ST revealed the presence of several plasmid genes such as *ColKP3*, *ColRNAI*, *ColBS512*, *ColpVC*, *IncFIA*, *IncFIB*, *IncFII*, *IncI1*, and *IncX3*. Among the genes responsible for ESBL, this isolate showed the presence of *bla*_CTX-M-15_, *bla*EC-18, and *bla*_TEM-1 _genes, among others.

ST-156 (O54:H28)

In the present study, this ST revealed the presence of plasmid genes such as *IncFIB *and *IncFII*. Among the genes responsible for ESBL, this isolate showed the presence of *bla*_CMY-42_, *bla*_EC-15_, and *bla*_OXA-181_ genes, among others.

ST-2851 (O100:H18)

This ST demonstrated the presence of *bla*_CTX-M-15_,* bla*_CMY-42_, *bla*_NDM-5_, *bla*_EC_, and *bla*_TEM-1_ genes, among others. The plasmid replicons identified included *Col (MG828)*, *Col (pHAD28)*,* IncF1A*, *IncF1B (pNDM-Mar)*, *IncFII*, *IncHI1B (pNDM-MAR)*, and *IncI (Gamma)*.

ST-827 (O4:H1)

In the present study, this ST revealed the presence of plasmid genes such as *IncB*/*O*/*K*/*Z*, *IncFIB (AP001918)*, and *IncFII*. The genes responsible for ESBL were not identified in this isolate.

ST-2006 (O9:H23)

This ST demonstrated the presence of *bla*_CTX-M-15_ and *bla*_EC_ genes, among others. The plasmid replicons identified included *Col156*, *ColRNAI*, *IncFII (pCoo)*, *IncFIB (AP001918)*, *IncI (Gamma)*, and *IncY*.

ST-448 (O188:H4)

*ColBS512*, *IncFIA*, *IncFIB*, and *IncFII *were the plasmid replicons identified in this ST. Only *bla*EC-16 was noticed that coded for cephalosporin resistance.

ST-8881 (O153:H6)

In the present study, this ST revealed the presence of several plasmid genes such as *IncFIA*, *IncFIB (AP001918)*, and *IncFII (pRSB107)*. Among the genes responsible for ESBL, this isolate showed the presence of *bla*_CTX-M-15_, *bla*EC, *bla*_OXA_, and *bla*_TEM-1_ genes, among others.

ST-38 (O11:H30)

In the present study, this ST revealed the presence of several plasmid genes such as *IncFIB*, *IncFII*, and *IncX4*. Among the genes responsible for ESBL, this isolate showed the presence of *bla*_CTX-M-15_, *bla*EC-8, *bla*_OXA-1_, and *bla*_TEM-1_ genes, among others.

Single Locus Variant of ST-14 (O75:H5)

In the present study, this ST revealed the presence of several plasmid genes such as *Col156*, *ColRNAI*, *ColBS512*, *IncFIA*, *IncFIB*, *IncFII*, and *IncI1*. Among the genes responsible for ESBL, this isolate showed the presence of *bla*_CMY-2_ and *bla*EC-5 genes, among others.

Study limitations

This study analyzed a few *E*. *coli *strains isolated from patients with UTIs admitted to a tertiary care teaching hospital. The study also did not compare the phenotypic resistance characteristics of the individual isolates with the genes identified through WGS. Besides, the study did not carry out phylogenetic analysis and clade categorization/identification of the isolates. This study did not explore the carriage of resistance and virulence genes on specific plasmid replicons and their origins.

## Conclusions

The results of this study demonstrate that *E*. *coli *isolated from people suffering from UTIs carry several genes coding for drug resistance and other genes for virulence. This could enable them to gain MDR and become invasive, thereby contributing to severe morbidity and mortality among the affected population. Additionally, the *E*. *coli *analyzed in this study demonstrated plasmid genes that have an increased potential to carry drug-resistant genes, which can be transmitted to other strains through horizontal or vertical routes. The results of this study emphasize the role of WGS in understanding the genetic characteristics of bacteria and predicting the emergence of antimicrobial resistance. Data generated from such studies require elaborate analysis before being applied to make decisions regarding the use of antimicrobial agents, thereby preventing future emergences of MDR strains. Further, these decisions can minimize treatment failures stemming from infections caused by the MDR bacterial strains.

## References

[1] Haney EF, Hancock RE (2022). Addressing antibiotic failure-beyond genetically encoded antimicrobial resistance. Front Drug Discov.

[2] Zhou S, Barbosa C, Woods RJ (2020). Why is preventing antibiotic resistance so hard? Analysis of failed resistance management. Evol Med Public Health.

[3] Mueller M, Tainter CR (2024). Escherichia coli infection. https://www.ncbi.nlm.nih.gov/books/NBK564298/.

[4] Giuliano C, Patel CR, Kale-Pradhan PB (2019). A guide to bacterial culture identification and results interpretation. P T.

[5] Baron EJ, Finegold SM (1998). Overview of conventional methods for bacterial identification. Bailey and Scott’s diagnostic microbiology.

[6] Collee JG, Miles RS, Watt B (1996). Tests for the identification of bacteria. Mackie & McCartney practical medical microbiology, 14th edition.

[7] Karah N, Rafei R, Elamin W, Ghazy A, Abbara A, Hamze M, Uhlin BE (2020). Guideline for urine culture and biochemical identification of bacterial urinary pathogens in low-resource settings. Diagnostics (Basel).

[8] (2020). Performance standards for antimicrobial susceptibility testing, 30th edition. https://www.nih.org.pk/wp-content/uploads/2021/02/CLSI-2020.pdf.

[9] Bankevich A, Nurk S, Antipov D (2012). SPAdes: a new genome assembly algorithm and its applications to single-cell sequencing. J Comput Biol.

[10] Seemann T (2014). Prokka: rapid prokaryotic genome annotation. Bioinformatics.

[11] Low AJ, Koziol AG, Manninger PA, Blais B, Carrillo CD (2019). ConFindr: rapid detection of intraspecies and cross-species contamination in bacterial whole-genome sequence data. PeerJ.

[12] Hunt M, Mather AE, Sánchez-Busó L, Page AJ, Parkhill J, Keane JA, Harris SR (2017). ARIBA: rapid antimicrobial resistance genotyping directly from sequencing reads. Microb Genom.

[13] Jung Y, Han D (2022). BWA-MEME: BWA-MEM emulated with a machine learning approach. Bioinformatics.

[14] Marshall CR, Chowdhury S, Taft RJ (2020). Best practices for the analytical validation of clinical whole-genome sequencing intended for the diagnosis of germline disease. NPJ Genom Med.

[15] Ventola CL (2015). The antibiotic resistance crisis: part 1: causes and threats. P T.

[16] Magiorakos AP, Srinivasan A, Carey RB (2012). Multidrug-resistant, extensively drug-resistant and pandrug-resistant bacteria: an international expert proposal for interim standard definitions for acquired resistance. Clin Microbiol Infect.

[17] Friedman ND, Temkin E, Carmeli Y (2016). The negative impact of antibiotic resistance. Clin Microbiol Infect.

[18] Susić E (2004). [Mechanisms of resistance in Enterobacteriaceae towards beta-lactamase antibiotics]. Acta Med Croatica.

[19] Bush K, Bradford PA (2016). Β-lactams and β-lactamase inhibitors: an overview. Cold Spring Harb Perspect Med.

[20] Paterson DL, Bonomo RA (2005). Extended-spectrum beta-lactamases: a clinical update. Clin Microbiol Rev.

[21] Queenan AM, Bush K (2007). Carbapenemases: the versatile beta-lactamases. Clin Microbiol Rev.

[22] Cui X, Zhang H, Du H (2019). Carbapenemases in Enterobacteriaceae: detection and antimicrobial therapy. Front Microbiol.

[23] Sawa T, Kooguchi K, Moriyama K (2020). Molecular diversity of extended-spectrum β-lactamases and carbapenemases, and antimicrobial resistance. J Intensive Care.

[24] Mazumder R, Abdullah A, Ahmed D, Hussain A (2020). High prevalence of blaCTX-M-15 gene among extended-spectrum β-lactamase-producing Escherichia coli isolates causing extraintestinal infections in Bangladesh. Antibiotics (Basel).

[25] Singh NS, Singhal N, Virdi JS (2018). Genetic environment of bla(TEM-1), bla(CTX-M-15), bla(CMY-42) and characterization of integrons of Escherichia coli isolated from an Indian urban aquatic environment. Front Microbiol.

[26] Zhang L, Lü X, Zong Z (2013). The emergence of blaCTX-M-15-carrying Escherichia coli of ST131 and new sequence types in Western China. Ann Clin Microbiol Antimicrob.

[27] Sun P, Xia W, Liu G (2019). Characterization of bla (NDM-5)-positive Escherichia coli prevalent in a university hospital in eastern China. Infect Drug Resist.

[28] Devanga Ragupathi NK, Veeraraghavan B, Muthuirulandi Sethuvel DP (2020). First Indian report on genome-wide comparison of multidrug-resistant Escherichia coli from blood stream infections. PLoS One.

[29] Nicolas-Chanoine MH, Bertrand X, Madec JY (2014). Escherichia coli ST131, an intriguing clonal group. Clin Microbiol Rev.

[30] Forde BM, Roberts LW, Phan MD (2019). Population dynamics of an Escherichia coli ST131 lineage during recurrent urinary tract infection. Nat Commun.

[31] Shen P, Yi M, Fu Y, Ruan Z, Du X, Yu Y, Xie X (2017). Detection of an Escherichia coli sequence type 167 strain with two tandem copies of blaNDM-1 in the chromosome. J Clin Microbiol.

[32] Garcia-Fernandez A, Villa L, Bibbolino G (2020). Novel insights and features of the NDM-5-producing Escherichia coli sequence type 167 high-risk clone. mSphere.

[33] Alba P, Taddei R, Cordaro G (2021). Carbapenemase IncF-borne bla(NDM-5) gene in the E. coli ST167 high-risk clone from canine clinical infection, Italy. Vet Microbiol.

[34] Pan S, Liu S, Tai S, Yu J, Yuan E, Duan Y (2023). Genomic analysis of an Escherichia coli sequence type 167 isolate harboring a multidrug-resistant conjugative plasmid, suggesting the potential transmission of the type strains from animals to humans. Infect Drug Resist.

[35] Farzana R, Jones LS, Rahman MA (2023). Genomic insights into the mechanism of carbapenem resistance dissemination in Enterobacterales from a tertiary public heath setting in South Asia. Clin Infect Dis.

[36] Zou H, Jia X, Liu H, Li S, Wu X, Huang S (2020). Emergence of NDM-5-producing Escherichia coli in a teaching hospital in Chongqing, China: INCF-type plasmids may contribute to the prevalence of bla (NDM-) (5). Front Microbiol.

[37] Flament-Simon SC, Toro M, García V (2020). Molecular characteristics of extraintestinal pathogenic E. coli (ExPEC), uropathogenic E. coli (UPEC), and multidrug resistant E. coli isolated from healthy dogs in Spain. Whole genome sequencing of canine ST372 isolates and comparison with human isolates causing extraintestinal infections. Microorganisms.

[38] Roer L, Overballe-Petersen S, Hansen F (2018). Escherichia coli sequence type 410 is causing new international high-risk clones. mSphere.

[39] Lee M, Choi TJ (2020). Species transferability of Klebsiella pneumoniae carbapenemase-2 isolated from a high-risk clone of Escherichia coli ST410. J Microbiol Biotechnol.

[40] Ewbank AC, Fuentes-Castillo D, Sacristán C (2022). World Health Organization critical priority Escherichia coli clone ST648 in magnificent frigatebird (Fregata magnificens) of an uninhabited insular environment. Front Microbiol.

[41] Ewers C, Bethe A, Stamm I (2014). CTX-M-15-D-ST648 Escherichia coli from companion animals and horses: another pandemic clone combining multiresistance and extraintestinal virulence?. J Antimicrob Chemother.

[42] Campos AC, Andrade NL, Ferdous M (2018). Comprehensive molecular characterization of Escherichia coli isolates from urine samples of hospitalized patients in Rio de Janeiro, Brazil. Front Microbiol.

[43] Paulshus E, Thorell K, Guzman-Otazo J (2019). Repeated isolation of extended-spectrum-β-lactamase-positive Escherichia coli sequence types 648 and 131 from community wastewater indicates that sewage systems are important sources of emerging clones of antibiotic-resistant bacteria. Antimicrob Agents Chemother.

[44] Medugu N, Aworh MK, Iregbu K (2022). Molecular characterization of multi drug resistant Escherichia coli isolates at a tertiary hospital in Abuja, Nigeria. Sci Rep.

[45] Day MJ, Hopkins KL, Wareham DW (2019). Extended-spectrum β-lactamase-producing Escherichia coli in human-derived and foodchain-derived samples from England, Wales, and Scotland: an epidemiological surveillance and typing study. Lancet Infect Dis.

[46] Schaufler K, Semmler T, Wieler LH (2019). Genomic and functional analysis of emerging virulent and multidrug-resistant Escherichia coli lineage sequence type 648. Antimicrob Agents Chemother.

[47] Roy Chowdhury P, McKinnon J, Liu M, Djordjevic SP (2018). Multidrug resistant uropathogenic Escherichia coli ST405 with a novel, composite IS26 transposon in a unique chromosomal location. Front Microbiol.

[48] Sumbana JJ, Santona A, Fiamma M (2021). Extraintestinal pathogenic Escherichia coli ST405 isolate coharboring blaNDM-5 and blaCTXM-15: a new threat in Mozambique. Microb Drug Resist.

[49] Gaviria LP, Montsant L, Azuaje C (2022). A descriptive analysis of urinary ESBL-producing-Escherichia coli in Cerdanya Hospital. Microorganisms.

